# The Uniform System of Hospital Accounts

**Published:** 1907-02-09

**Authors:** Arthur R. White, A. Herbert Webber

**Affiliations:** Hon. Treasurer. Clapham General and Provident Dispensary; Hon. Secretary, Clapham General and Provident Dispensary


### Editor's Letter-Box.

[Our Correspondents are reminded that prolixity is a great bar to publication, and that brevity of style and conciseness of statement greatly facilitate early insertion.]

### THE UNIFORM SYSTEM OF HOSPITAL ACCOUNTS.

*To the Editor of* The Hospital.

Sir,—As regards the amended uniform system of hospital accounts, issued on December 1, 1906, by the three great hospital funds, in the form of a red-covered shilling pamphlet, we desire to submit a few observations.

1. We assume that the new system cannot apply to the metropolitan dispensaries—inasmuch as the Royal Fund has refused to recognise the public services of the dispensaries, and as the word dispensary does not, so far as we can see, occur in the said pamphlet, and the directing words on page 2 of same refer only to the changes which have been determined upon and which all hospitals applying for grants from either of the funds in future are requested to observe.

2. Whatever costly or complicated system of accounts may be needed for the hospitals, it would appear to us most unfair to inflict same upon the poor dispensaries, and we venture to express a hope that the dispensaries may be allowed to adopt the new income and expenditure account set out on pages 8 and 9 of the said pamphlet, as a cash account, and with the addition of the balances at the beginning and end of the financial year, without which we venture to affirm that no subscriber can form an accurate idea of the financial position of the institution to which he is subscribing.

3. The complicated balance-sheet on page 10 of the pamphlet is obviously unsuited to the simple requirements of a dispensary.

4. In any case we are assuming that as the alterations are to date from January 1, 1907 (page 2 of pamphlet), they cannot be held to apply to the accounts for the year ending December 31, 1906.

We are, sir, yours obediently,

Arthur R. White,
Hon. Treasurer.

A. Herbert Webber,
Hon. Secretary,

Clapham General and Provident Dispensary.

